# Reward-based learning for virtual neurorobotics through emotional speech processing

**DOI:** 10.3389/fnbot.2013.00008

**Published:** 2013-04-29

**Authors:** Laurence C. Jayet Bray, Gareth B. Ferneyhough, Emily R. Barker, Corey M. Thibeault, Frederick C. Harris

**Affiliations:** ^1^Department of Computer Science and Engineering, University of NevadaReno, NV, USA; ^2^Department of Bioengineering, George Mason UniversityFairfax, VA, USA; ^3^HRL Laboratories, LLCMalibu, CA, USA

**Keywords:** emotional speech processing, reward-based learning, virtual neurorobotics, biological computational model

## Abstract

Reward-based learning can easily be applied to real life with a prevalence in children teaching methods. It also allows machines and software agents to automatically determine the ideal behavior from a simple reward feedback (e.g., encouragement) to maximize their performance. Advancements in affective computing, especially emotional speech processing (ESP) have allowed for more natural interaction between humans and robots. Our research focuses on integrating a novel ESP system in a relevant virtual neurorobotic (VNR) application. We created an emotional speech classifier that successfully distinguished happy and utterances. The accuracy of the system was 95.3 and 98.7% during the offline mode (using an emotional speech database) and the live mode (using live recordings), respectively. It was then integrated in a neurorobotic scenario, where a virtual neurorobot had to learn a simple exercise through reward-based learning. If the correct decision was made the robot received a spoken reward, which in turn stimulated synapses (in our simulated model) undergoing spike-timing dependent plasticity (STDP) and reinforced the corresponding neural pathways. Both our ESP and neurorobotic systems allowed our neurorobot to successfully and consistently learn the exercise. The integration of ESP in real-time computational neuroscience architecture is a first step toward the combination of human emotions and virtual neurorobotics.

## 1. Introduction

How does speech portray emotions? Many of our social cues and communication skills rely on emotional speech, but it is a challenging process to study. Affective computing, especially emotional speech processing (ESP) has helped elucidate the importance of human emotions. It is basically described as applying human like emotional effects to artificially produced speech. Speech contains acoustic features that vary with the speaker's affective state, and the ability to interpret these communication signals (e.g., emotions) affects social interaction (Warren et al., 2006). Humans also perceive how emotional environmental cues such as fear or anger indicate danger (Kanske and Hasting, 2010) and keep them fit for survival.

At the physiological level, speech is processed in specialized brain regions in the upper portion of the superior temporal sulcus, which is one of the voice-selective areas of the auditory cortex (Grossmann et al., 2010). These areas in monkeys and humans have been thought to provide social information to sensory systems. Recent studies on macaque monkeys have revealed they have a region in the superior temporal plane selective to speech similar to humans (Belin et al., 2000, 2004). These studies suggest that recognition of speech within species is an evolutionarily conserved brain function in primates and is independent of language (Petkov et al., 2008, 2009). Therefore, language requires more than simply linguistic information. Other studies in behavioral biology, psychology, and speech and communication sciences have suggested that many emotional states are communicated by specific acoustic characteristics of the speaker. Evidence reveals that listeners attend to changes in voice quality, articulation, pitch, and loudness to understand the speaker's emotion (Banse and Scherer, 1996). Emotions that are the most distinct in humans are anger, disgust, fear, joy, sadness, and surprise (El Ayadi et al., 2011).

As part of emotional processing, emotional speech recognition is a relatively recent research field, which is defined as extracting the emotional state of a speaker from her or his speech (El Ayadi et al., 2011). Automatic recognition of emotions from modalities such as speech has acquired expanding interest within the area of human-machine interaction research (Fu et al., 2010). Such emotional speech recognition is essential for facilitating realistic communication between robots and humans. Service robots are being designed to help humans with difficult or time-consuming tasks or help those with disabilities (Severinson-Eklundh et al., 2003). Appropriate communication allows robots to share human knowledge, and can potentially use human recognition capabilities to complete complex tasks (Ghidary et al., 2002). Thus, it will be important for future robotics to be able to understand emotion in speech in order to complete such tasks.

Biological-inspired human-robot interactions have become increasingly important as robots fascinate many researchers and become more common in our daily activities. For the past couple of years, we have worked on machine learning systems, and we developed a Virtual Neurorobotic (VNR) loop, which focuses on the coupling of neural systems with some form of physical actuation. This is based around the interoperability of a neural model, a virtual robotic avatar and a human participant (Goodman et al., 2007, 2008). Under all but the most basic scenarios this interoperability is accomplished through an organized network communication system (Thibeault et al., 2010b, 2012).

This paper provides an introduction to affective computing and emotional speech processing combined with one application of real-time virtual neurorobotics. We use our VNR to describe how our emotional speech system can be successfully used to reinforce learning and allow a neurorobot to make ideal choices based on visual cues.

## 2. Affective computing

The curious nature of human emotions has been the subject of much research and philosophical debate. Why do humans have emotions, and what role do they have in human cognition and behavior? During the cognitive revolution that began in the second half of the twentieth century, the lingering influence of behaviorism helped downplay the role of emotion to little more than a side effect from instinctual and learned behavior (Hudlicka, 2003).

Recently, advancements in neuroscience and psychology have helped elevate the importance of emotion; within the last decade or so, research has shown that emotion plays a crucial role in human intelligence, including planning and decision making of all levels (Hudlicka, 2003; Picard, 2003). This renewed interest in emotional research has led to the birth of a growing research field, affective computing. Rosalind Picard's paper, *Affective Computing: Challenges* gave the field its name (Picard, 2003). In her paper, Picard discusses the three main areas of affective computing: emotional sensing and recognition, affect modeling, and emotion expression.

Several researchers have attempted to create emotionally intelligent robots. Perhaps the most famous is Kismet, an infant-like robotic creature developed at MIT (Breazeal and Aryananda, 2002). Kismet responds to the emotional state (typically acted) of its “caregiver” by analyzing the caregiver's speech in real-time. The system extracts statistics on the caregiver's voice pitch and energy, and classifies the underlying emotion using a Gaussian mixture model classifier. The robot responds to the caregiver's emotional intent by changing its facial expression. Naive test subjects were chosen to interact with the robot and many felt a strong emotional response while interacting with it, especially when Kismet showed sadness after being prohibited by the human. Kismet successfully shows that robots can be designed to react to human emotions, and in turn, elicit an emotional response from the human as well.

Another empathetic android robot is BARTHOC, developed at Bielefeld University, Germany (Hegel et al., 2006). BARTHOC can be given several different appearances by changing the latex mask that composes its face and head. For many experiments, the robot is given the appearance of a small child via a latex mask, although its appearance is decidedly less “cute” than Kismet, due to the difficulty in creating a realistic looking android face. Like Kismet, BARTHOC mimics the emotion of the human interacting with it by changing its facial expression. The emotion of the human is determined using emotional speech processing. BARTHOC can distinguish and portray six emotional states: neutral, happy, fear, anger, disgust, surprise, and sad.

Both Kismet and BARTHOC can mimic human emotions by recognizing the emotional content in a human's speech. Our system aims to further these advancements by using human emotional content as a training mechanism for a virtual robot.

## 3. Theory behind emotional speech processing (ESP)

ESP systems (also called emotional speech recognition systems) attempt to determine the underlying emotion in human speech. Unlike normal speech recognition systems, most ESP systems do not extract lexical information, but instead classify the speaker's emotion without any regard to context. This is typically accomplished by extracting prosodic features for each word or phrase uttered by the speaker, generating statistics on these features, and classifying the feature vector using a supervised learning algorithm.

Although the accuracy of ESP systems is typically lower than other emotional classification methods involving facial imaging and physiological features, their recognition rates are similar to those of humans (Hudlicka, 2003). Furthermore, emotional speech recognition is less computationally expensive and less invasive than other methods, and remains a popular method for emotion detection, especially in live environments.

### 3.1. Features

There is currently little consensus on the best features for emotional speech recognition, however statistics on prosodic features, especially the fundamental frequency (pitch), are among the most common (Scherer et al., 1991; Dellaert et al., 1996; Oudeyer, 2003; Ververidis et al., 2004; Fu et al., 2010; Thibeault et al., 2010b; Koolagudi et al., 2011; Tahon et al., 2011). Other prosodic features used for ESP include energy and duration (Batliner et al., 2006). In addition to prosody, other common features include spectral features such as Mel-frequency cepstral coefficients (MFCCs), and non-linear Teager energy based features. In order to form a “good“ feature vector, ESP systems extract several statistical quantities from each feature contour such as the “mean, median, standard deviation, maximum, minimum, range, linear regression coefficients, 4th order Legendre parameters, vibrations, mean of first difference, mean of the absolute of the first difference, jitter, and ratio of the sample number of the up-slope to that of the down-slope of the pitch contour” (El Ayadi et al., 2011). By varying the number of features, and the statistics on each feature, ESP systems can have feature vectors of lengths ranging from 12 (Breazeal and Aryananda, 2002) to 988 (Eyben et al., 2009). To improve classification time and accuracy, several studies begin with large feature sets and then select the best features using exhaustive, sequential, or random searches (Fu et al., 2010).

### 3.2. Fundamental frequency detection

The fundamental frequency (*F*_0_) of a voiced speech is typically defined as the rate of vibration of the vocal folds (de Cheveigné and Kawahara, 2002). Generally, the pitch humans perceive when someone is talking or singing is equivalent to the fundamental frequency, and ranges from about 40 to 600 Hz (Huang et al., 2001). We will therefore refer to the fundamental frequency simply as “pitch”, and methods to determine *F*_0_ as “pitch detection algorithms.” Frequency-domain pitch detection approaches usually utilize the Fast Fourier Transform (FFT) to convert the signal to the frequency spectrum. This allows for polyphonic detection. Time-domain approaches, such as autocorrelation are typically less computationally expensive, but may be prone to errors and octave jumps, especially due to noise. As a method, robust algorithm for pitch tracking (RAPT) (Talkin, 1995) is a pitch tracking algorithm that attempts to return a smooth pitch contour, without the undesirable octave jumps and false detection problems present in the basic auto-correlation method. RAPT operates on two versions of the input signal, one at the original sample rate, and one at a significantly reduced rate. The algorithm first computes the normalized cross-correlation (NCFF) of a low-sample signal and records the locations of the local maxima. Next, NCFF is performed on the higher sample-rate signal in the vicinity of the peaks found in the previous step. This generates a list of several *F*_0_ candidates for the input frame. Finally, dynamic programming is used to select the best *F*_0_ candidates over the entire window.

### 3.3. Classifiers

After a feature vector has been created, it must be classified in order to determine its emotional class. A number of classifiers have been used in ESP systems, including hidden Markov models (HMM), Gaussian mixture models (GMM), k-nearest neighbor (k-NN), support vector machines (SVM), artificial neural networks (ANN), and decision trees (El Ayadi et al., 2011). Different classifiers can perform better in different situations, which can have a significant effect of a system's classification accuracy (El Ayadi et al., 2011). Therefore, it is important for the researcher to chose a classifier carefully, taking into account accuracy as well as computational requirements.

### 3.4. Databases

It can be difficult to compare the classification accuracies reported by different researchers due to the variety in emotional speech databases used. The Berlin emotional speech database (Burkhardt et al., 2005) contains recordings performed by professional actors in a noise-free environment, while (Morrison et al., 2007) provides actual recordings from call centers. Naturally, both humans and computers attain higher recognition accuracy on databases containing low-noise, acted recordings.

## 4. Virtual neurorobotics (VNR)

VNR aims to develop combinations of biologically realistic neural simulations with robotic agents and human participants in closed-loop configurations (Thibeault et al., 2010b). As described by our previous studies by Goodman et al. (2007, 2008), we define VNR as follows: a computer-facilitated behavioral loop wherein a human interacts with a projected robot that meets five criteria: the robot is sufficiently embodied for the human to tentatively accept the robot as a social partner; the loop operates in real time, with no pre-specified parcellation into receptive and responsive time windows; the cognitive control is a neuromorphic brain emulation using our NeoCortical simulator (NCS) and incorporating realistic neuronal dynamics whose time constants reflect synaptic activation, membrane and circuitry properties, and most importantly learning; the neuromorphic architecture is expandable to progressively larger scale and complexity to track brain development; and the neuromorphic architecture can potentially provide circuitry underlying intrinsic motivation and intentionality, which physiologically is best described as emotional rather than rule-based drive.

NCS (Drewes, 2005; Wilson et al., 2005; Brette et al., 2007; Drewes et al., 2009; Jayet Bray et al., 2010) is a neural simulator that can model integrate-and-fire neurons with conductance-based synapses. It uses two clusters: four SUN 4600 machines (16-processors each) connected via Infiniband with 192 GB RAM per machine, 24 Terabytes of disk storage; and 208 Opteron cores, 416 GB RAM, and more than a Terabyte of disk storage. Note: for more information on NCS equations and related publications, please go to: www.cse.unr.edu/brain/publications.

**Figure 1:**
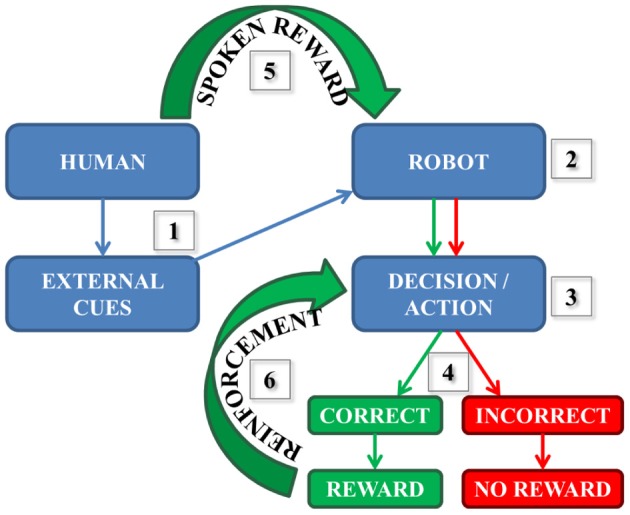
**Simplified reward-based learning scheme during human-robot interaction.** (1) The external cue is presented by the human to the robot; (2) The information is seen and processed by the robot; (3) The decision and the related action are performed; (4) The robot chose the ball correctly or incorrectly; (5) The spoken reward (if correct action) is received by the robot (6) The reward reinforces Learning every time the decision is correct.

As a part of our neurorobotics, learning can be based on many different experiences including making correct decisions and consequently being rewarded. As illustrated in Figure 1: (1) a human participant presents the robot with one external cue at a time. The robot sees and then processes the information (2), then a decision followed by an action associated with the initial cue is made (3). Then, there are two possible scenarios (4): If the decision/action is incorrect, then the robot does not receive any reward. However, if the decision is correct it does receive a reward (e.g., hears positive speech) by the human. In our correct case, the reward stimulates synapses (in our simulated model) that underwent spike-timing dependent plasticity (STDP) described by several studies (Zhang et al., 1998; Song et al., 2000; Dan and Poo, 2004; Caporale and Dan, 2008; Markram et al., 2011) as:

(1) $$W(\Delta t)=\{\begin{array}{ll} A_{+}\exp\text{​}\left(\frac{\Delta t}{\tau_{+}}\right) & \text{if }(\Delta t)<0\\ -A_{-}\exp\text{​}\left(\frac{-\Delta t}{\tau_{-}}\right) & \text{if }(\Delta t)\geq 0 \end{array}$$

where A is the maximum amount of synaptic modification; Δ*t* is the positive or the negative window; and τ is the positive or the negative decay constant.

Every time the robot receives a spoken reward (5), the neural pathway corresponding to the correct decision and the action is reinforced (6) until completely learned.

The integration of ESP in real-time computational neuroscience architecture is a first step toward the combination of human emotions and virtual neurorobotics. It was first described in our preliminary study by Thibeault et al. (2010b), and it is now being improved and further implemented in one of our neurorobotic applications. The improvements consisted on making the system a stand alone C++ application using a different classification and an ameliorated feature extraction method as described in Section 5.

## 5. Methods

### 5.1. Human emotional speech classification

To provide a benchmark for our emotional speech classification system, we conducted a human trial in which seven individuals were asked to classify 40 random utterances (sentences) from the Berlin emotional speech database from four emotional classes: happy, sad, anger, and fear. An even amount of samples (10) was randomly played for each of the four emotions. Therefore, a total of 280 samples (70 for each emotion) were classified and displayed in a confusion matrix (Table 1). All the samples in the database were in German and the humans classifying the samples only spoke English. This allowed the listeners to only base their classifications on the prosody only, rather than the meaning of the words.

**Table 1:** **Human classification confusion matrix**.

**Category**	**Anger**	**Fear**	**Happy**	**Sad**	**Error**
Anger	62	3	5	0	11.4%
Fear	5	62	1	2	11.4%
Happy	5	8	56	1	20.0%
Sad	0	1	1	68	2.9%
Average error					11.4%

### 5.2. Emotional speech recognition system

Our emotional speech classification system operated in real-time by extracting several prosodic features for each utterance, and classifying them using the support vector machine library, libSVM (Chang and Lin, 2011) with the Radial Basis Function (RBF) kernel. This classifier was chosen because of its high accuracy for emotional speech classification tasks (El Ayadi et al., 2011).

To form the prosodic feature vector for each utterance, the pitch for each window was determined using RAPT (Talkin, 1995), as described in Section 3. The window size and overlap were 3361 and 2880 frames long, respectively. These values were suggested by the RAPT algorithm for our system's particular sample rate of 16 KHz. In addition to the pitch, RAPT also returned the signal energy for each window. If the energy was above a dynamic threshold, RAPT assumed that the speaker was talking. In this case, the energy and pitch for that window were saved. If the energy was too low, the speaker assumed to be silent and the window was discarded.

**Figure 2:**
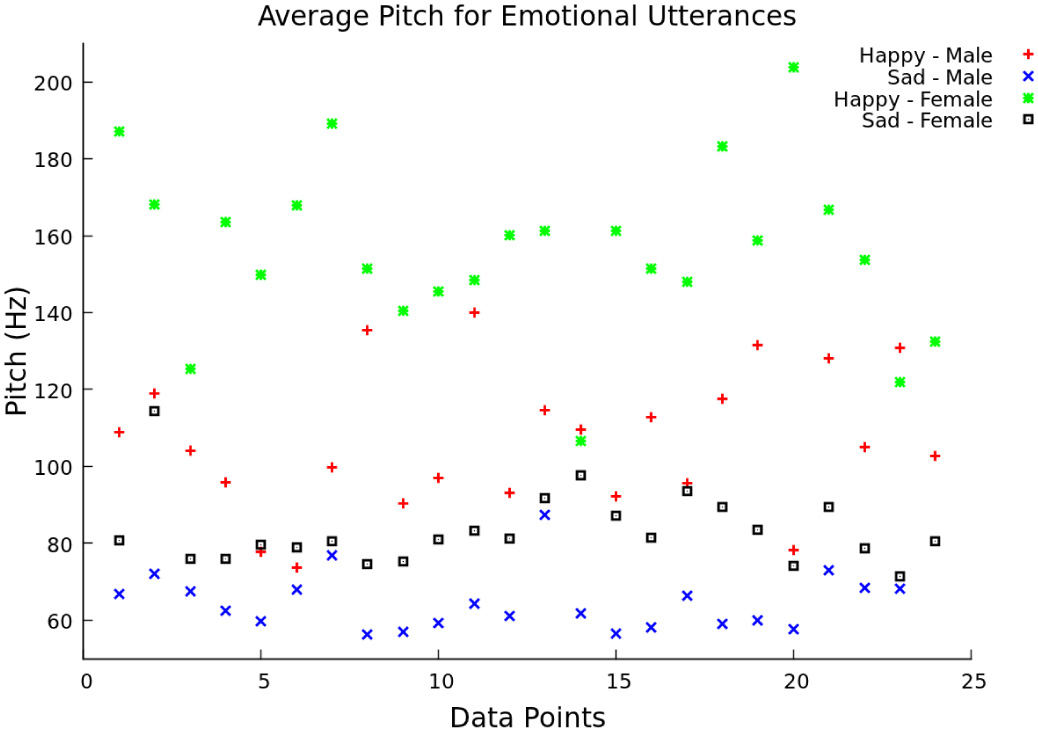
**Average pitch between two emotional utterances.** The average pitch (Hz) is shown over 14 data points for both “happy” (female voice—green and male voice—red) and “sad” (female voice—black and male voice—blue) utterances.

The system continued saving pitch and energy values for each window until a two second break in speech was was detected. This corresponded to the end of a utterance. After the end of an utterance, the feature vector was formed by calculating the mean, minimum, maximum, and range of the pitch values over the utterance. In addition to these four values, the feature vector also contained the mean speech energy during the voiced regions. In testing mode, the feature vector was then scaled and classified using libSVM. Before the system could classify emotions, it had to be trained (training mode). Features were extracted for 33% (offline) and 50% (live) of utterances, and they were given the appropriate emotion class labels. When the desired number of utterances was processed by the system, the feature vectors were scaled and used to create a libSVM model. The model file and scaling parameters were saved and used to classify the feature vectors in testing mode. To show the difference between pitch and emotion, the average pitch over 23 utterances was graphed comparing “happy” and “sad” emotion for both male and female speakers (Figure 2). This illustrates how the different pitch measurements change with respect to emotion and gender, as supported by Ververidis and Kotropoulos (2006).

There were two different experiments conducted to evaluate the classification accuracy of our system. The JACK Audio Connection Kit (Davis, 2013) was used to connect audio to the system, either from a separate audio player (offline mode) or the microphone (live mode). In the offline mode, the same pre-recorded samples (23 “happy” and “sad” utterances for both male and female speakers) from the Berlin emotional speech database were used, which gave a total of 92 samples. In the live mode, four humans recorded samples at 16 KHz from a list of 10 neutral phrase samples. The following samples were recorded: “Look Jack, the ball is blue,” “The ball is red,” “You turned left toward the library,” “Jack, you turned right toward the museum,” “You pointed to the blue color,” “You pointed to the red color, Jack,” “Jack, you went over there,” “Look what you've done,” “Jack gave the rattle to his mom,” “Jack kept the rattle for himself.” Each sample was recorded twice with both “happy” and “sad” utterances giving a total of 160 phrase samples. For both experiments, the results were represented as confusion matrices distinguishing “happy” and “sad” utterances from both female and male speakers. These showed the accuracy of the system (in terms of % error) for both live and offline modes (Tables 2, 3).

**Table 2:** **Offline Mode Recognition confusion matrix**.

**Category**	**Happy-M**	**Sad-M**	**Happy-F**	**Sad-F**	**Error**
Happy-M	16	0	0	0	0.0%
Sad-M	2	13	0	0	13.3%
Happy-F	0	0	17	1	5.6%
Sad-F	0	0	0	12	0.0%
Average error					4.7%

**Table 3:** **Live Mode Recognition confusion matrix**.

**Category**	**Happy-M**	**Sad-M**	**Happy-F**	**Sad-F**	**Error**
Happy-M	22	0	0	0	0.0%
Sad-M	0	16	0	0	0.0%
Happy-F	0	0	19	1	5.0%
Sad-F	0	0	0	19	0.0%
Average error					1.3%

### 5.3. A virtual neurorobotic application

Our virtual neurorobotic loop used a virtual neurorobot as a remote agent, and the interaction between a camera and ESP. The design used in this project as well as the basic software engineering behind its implementation was further described in our previous research by Thibeault et al. (2012).

**Figure 3:**
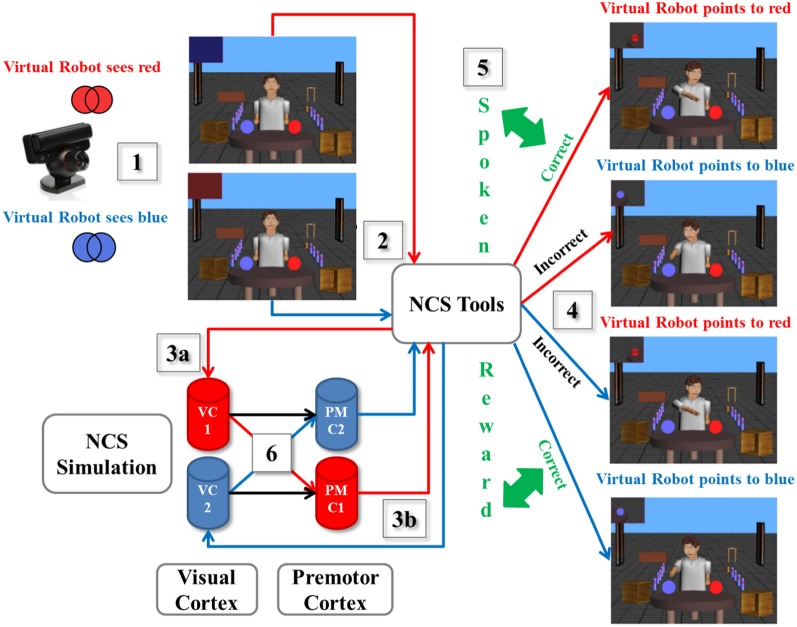
**A scenario of the virtual neurorobotic loop.** (1) The external cue (red or blue card) is presented by the human to the neurorobot (via the camera); (2) The Information is processed by the neurorobot and is sent to NCSTools; (3a) NCSTools stimulates the appropriate regions of the modeled visual cortex; (3b) NCSTools server monitores the neurorobot and creates the appropriate stimulus to send to proprioceptive feedback and premotor movements; (4) This loop of events initiates the appropriate pointing motion/action toward a colored ball; (5) After the robot has pointed to the correct or incorrect colored ball, the human participant responded with a “happy” or “sad” spoken phrase. This was processed by the ESP which determined whether the participant encouraged or discouraged the action of the virtual neurorobot; (6) The output of the ESP was coupled with the neural model via NCSTools. This reward stimulus was injected into groups of neurons (VC1 and VC2) to stimulate the corresponding synapses (to PMC1 and PMC2) that underwent STDP, which reinforced or depressed learning through time.

As a scenario example described in Figure 3, we designed an experiment using a spoken reward through ESP as reward-based learning. (1) A human presented a card with either a printed blue or red pattern to the neurorobot via the camera, which captured the image from the user and calculated the dominant color. (2) The information was processed by the virtual neurorobot, which was sent as the defined plain text statement (“saw red” or “saw blue”) to NCSTools through the server interface (Thibeault et al., 2012). (3a) The configuration of NCSTools stimulated the appropriate regions of the remote NCS Model through the NCS network interface (Jayet Bray et al., 2012). Images were then processed and respective values were sent to simulated visual pathways (Thibeault et al., 2010a). (3b) The NCSTools server monitored the neurorobot and created the appropriate stimulus to send to proprioceptive feedback and premotor movements. The NCSTools software then received spiking information from the premotor region of the neural simulation. Such activity in the two premotor regions were monitored, and then compared as the stimulation progressed. The appropriate command was finally sent to the neurorobot once a configured threshold was reached (Anumandla et al., 2011; Jayet Bray et al., 2012). (4) This loop of events initiated the appropriate pointing motion/action toward a colored ball. (5) After the robot has pointed to the correct or incorrect colored ball, the human participant responded with a “happy” or “sad” spoken phrase. This was processed by the ESP which determined whether the participant encouraged or discouraged the action of the virtual neurorobot. (6) The output of the ESP was fed through NCS Tools to the neural model. This reward stimulus was injected into groups of neurons (VC1 and VC2) to stimulate the corresponding synapses (to PMC1 and PMC2) that underwent STDP, which reinforced learning through time.

The experiment started as follows. The human showed a colored card randomly to the neurorobot (via the camera). On the first few attempts, the neurorobot had an equal chance of answering correctly or incorrectly since it was not familiar with the exercise. During this learning period, every time it chose the correct (incorrect) colored ball the “happy” (“sad”) reward was given. It took about 4 to 5 trials for the neurorobot to fully learn the exercise. Once it was completely familiar with the drill, no more rewards were necessary, but it continued to correctly point to the right color for the rest of the experiment. Overall, as shown in Figure 3 there were four possible scenarios: when the robot was shown the red (blue) pattern and correctly pointed to the red (blue) ball to its left (right), the human provided a happy spoken response. However, if the neurorobot incorrectly pointed right (left) to the blue (red) ball, a sad spoken response was given to the neurorobot.

For this simple example the reward was provided by correlated inputs between the previously activated visual column and the correctly chosen premotor column as well as reward activated STDP. In this case the plasticity of the synaptic connections was enabled during reward input. While the correlated firing encouraged the facilitation of the synapses resulting in an overall average increase in synaptic efficacy. It is important to emphasis that this reward mechanism is independent of the ESP system. The emotional classification can be used to activate any reward, punishment or input stimulus to the neural model. More sophisticated reward mechanisms such as those described in Florian (2007); Izhikevich (2007); Frmaux et al. (2010); Friedrich et al. (2011); O'Brien and Srinivasa (2013) will be explored in the future.

## 6. Results

The results of our emotional speech classification system and its integration as a reward in a VNR scenario are presented below.

### 6.1. Human emotional speech classification performance

From the classification system, an English speaking human was able to classify German speakers' emotions (fear, anger, happy, and sad) with an accuracy of 88.6%, as shown in the confusion matrix in Table 1. The vertical category column represents the actual class (Berlin emotional speech database recordings) where the horizontal category row is the classification of the emotion by the human subjects. For instance, Out of the 70 German “happy” tones 56 were correctly classified and 14 were incorrectly interpreted as either “fear” or “anger.” Additionally, the confusion table showed that most of the error occurred when the listener distinguished between “anger” and “happy,” when listening to an angry emotion OR when the listener distinguished between “happy” and “fear,” when listening to a happy utterance. This occurred because the utterances between these two emotions had similar features. This confusion can be expected between “anger” or “fear,” and happy in similar systems. Therefore, the “happy” and “sad” emotions were chosen for our neurorobotic application below due to a classification accuracy of 98.6%.

### 6.2. Emotional speech recognition system performance

In Figure 2, the average pitch is represented for the two chosen emotional classes (happy and sad) between the male and female groups from the Berlin emotional speech database. The “happy” utterance had a higher pitch frequency than the “sad” one, especially with female speakers. The “sad” male utterance had the lowest average pitch frequency overall.

During the offline mode, 92 samples from the Berlin emotional speech database (Burkhardt et al., 2005) were used to train (31 samples) and test (61 samples) the system. As shown in Table 2, 33 phrase samples of the 34 total happy samples (male and female combined) were correctly classified as happy while one was classified incorrectly as sad, giving an error of 5.6%. Out of the 27 total sad phrase samples (male and female combined), 25 were classified correctly while two were incorrectly classified as happy, giving an error of 13.3%. If we separate the male and female results, all 16 of the happy male phrase samples were correctly classified as happy, giving a 0% error. All of the 12 sad female samples were also correctly classified as sad, giving an error of 0%. The overall average error for all 61 phrase samples was 4.7%, which corresponds to a system accuracy of 95.3%. Note: Approximately 33% of the total 160 samples were used to train the system.

During the live mode, 160 samples from live recordings were used to train (83 samples) and test (77 samples) the system. As shown in Table 3, 41 phrase samples of the 42 total happy samples were correctly classified as happy while 1 was classified incorrectly as sad, giving an error of 5%. Out of the 35 total sad phrase samples, none were classified incorrectly, giving an error of 0%. The overall average error for all 77 phrase samples was 1.3%, which corresponds to a system accuracy of 98.7%. Note: Approximately 50% of the total 160 samples were used to train the system.

### 6.3. Virtual neurorobotic and reward-based learning

**Figure 4:**
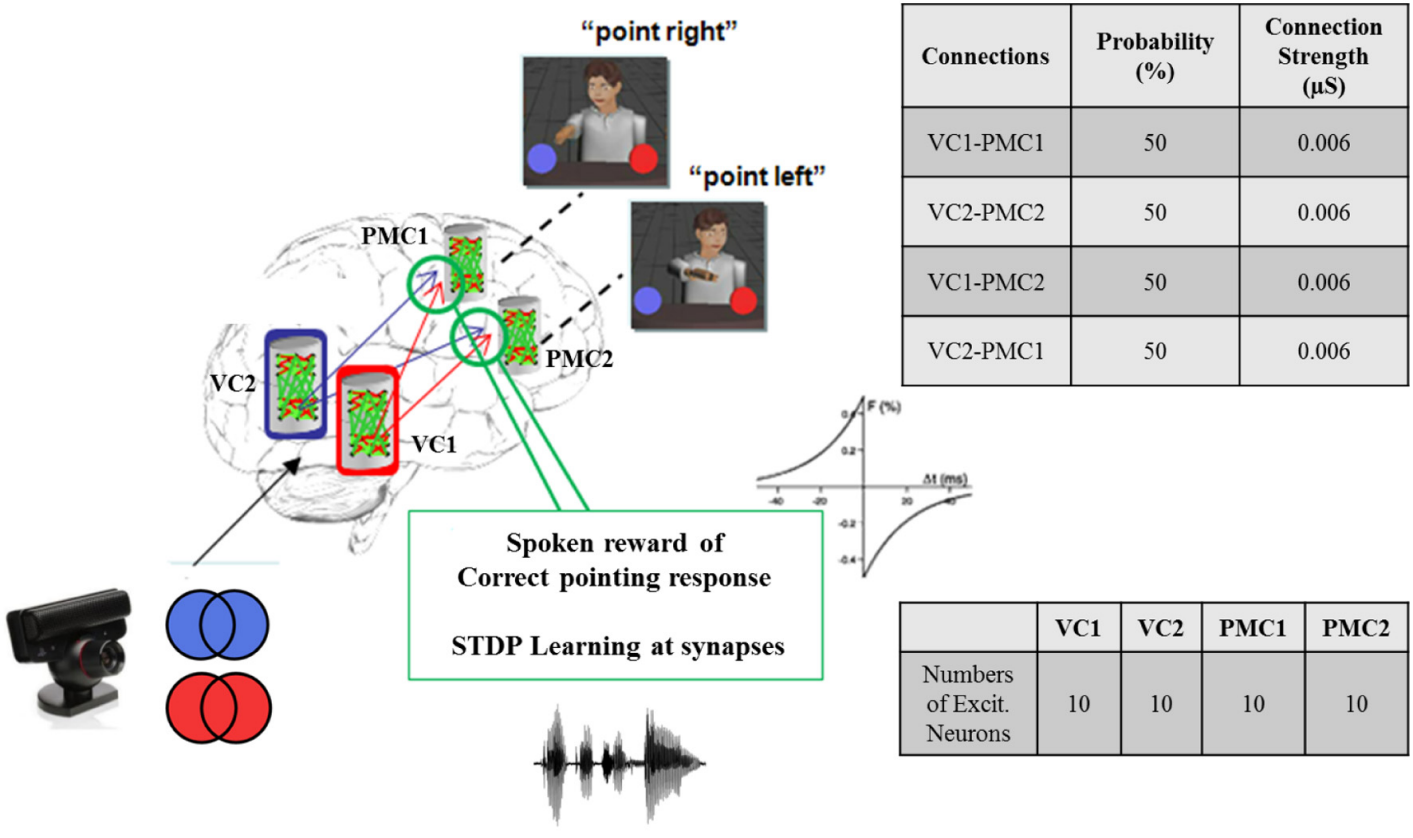
**Brain architecture in the virtual neurorobotic interface.** Simple spiking neuronal model composed of two areas: the visual cortex (VC) and the premotor cortex (PMC). Each area is divided into two columns VC1, VC2, and PMC1, PMC2 (10 neurons each), respectively. Each VC area has feedforward connections to both PMC regions (*P* = 50% and Max. conductance = 0.006 μS). The synaptic connections from VC1 (VC2) to PMC1 (PMC2) use STDP as a learning mechanism. As the red (blue) is presented to VC1 (VC2) the activity of the corresponding column increases, which make PMC1 (PMC2) fire. When the neurorobot points correctly to “red” (“blue”) a spoken reward is given, which stimulates the corresponding synapses VC1 → PMC1 (VC2 → PMC2).

In our neurorobotic application, the simple spiking neuron model used was an important aspect of the system, and it is illustrated in Figure 4. Once the camera captured either red or blue color, the visual information was processed and sent to NCSTools, as described in Section 5. The information was then converted and sent to NCS running on a remote computing cluster. The brain architecture was composed of two areas: the visual cortex (VC) and the premotor cortex (PMC) divided into four areas of 10 neurons: VC1, VC2, PMC1, and PMC2. Each VC column was connected to both PMC columns with a probability of connections of 50% and a connection strength of 0.006 μS. Only the connections from VC1 → PMC1 and VC2 → PMC2 had reinforcement learning synapses (positive STDP) where the other connections got depressed though time (negative STDP). Therefore, as the red pattern was presented VC1 activity increased, and consequently increased PMC1 firing. On the other hand, when the blue pattern was presented VC2 activity increased, and consequently increased PMC2 firing. As the simulation proceeded, the competing neural areas of visual and motor processing were monitored by NCSTools. The resulting activity was correlated with a pointing action to one of two colored balls that matched the color presented. After the robot pointed, a spoken reward was given to the robot if it pointed to the correct colored ball. The reward, analogous to a dopaminergic increase, resulted in an STDP dependent increase in synaptic efficacy (Zou and Destexhe, 2007). STDP was defined in Section 4, and in the model the maximum positive and negative amounts of synaptic modification (A) were 20 and 10 respectively; the positive and negative windows (Δ*t*) were 50 ms and 100 ms, respectively; and the positive and negative decay constants (τ) were both 5 ms.

**Figure 5:**
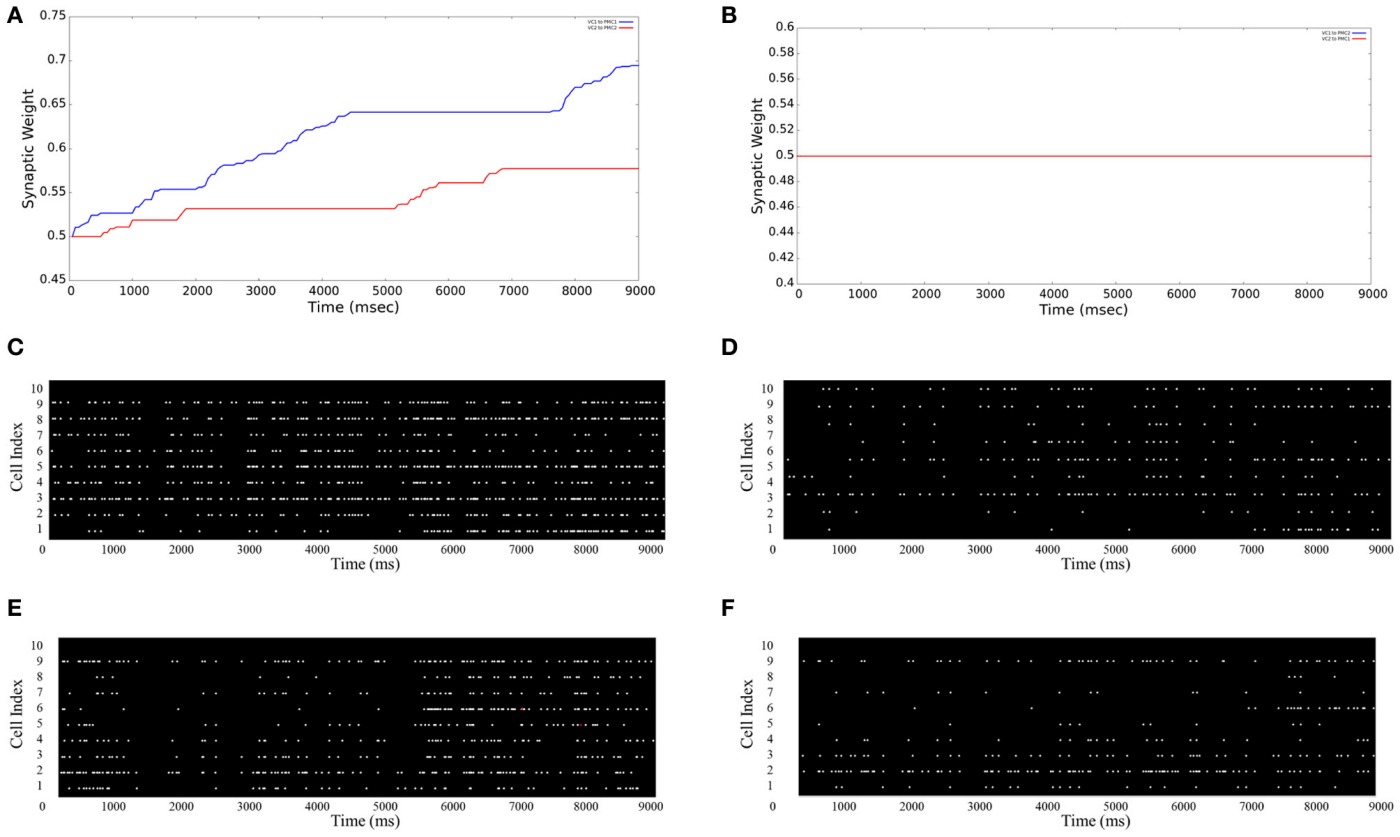
**Average synaptic weights and firing activities.** The GUI monitors the synaptic strengths over time as an average of all synapses between different neuron groups. **(A)** VC1 → PMC1 and VC2 → PMC2 synpases show an increase in their strengths due to STDP reinforcement; **(B)** VC1 → PMC2 and VC2 → PMC1 synpases show no increase in their strengths (note: the blue and red lines are superimposed); (**C** and **E**) PMC1 and PMC2 firing rates increase as learning occurs—the neurorobot makes correct choices; (**D** and **F**) PMC1 and PMC2 firing rates do not increase when the neurorobot is not rewarded—makes incorrect choices.

The Graphical User Interface (GUI) is an option given to users for visualizing aspects of the neural model in real-time. The user can specify each tab with the information of either: main window, stimulation input (VCs), and motor areas (PMCs). As shown in Figures 5A,B the average synaptic weight over the simulation time can be monitored. As an example for a 9 s simulation, Figure 5A shows both average synaptic weights increase between VC1 (VC2) and PMC1 (PMC2), which shows evidence that the neurorobot's correct decisions were reinforced over time. However, the average synaptic weights between “non-learning” synapses (VC1 to PMC2 and VC2 to PMC1) show no increase over time (Figure 5B). To support these results, the firing activity of both PMC1 and PMC2 is represented in (Figures 5C–F). They increase as reinforcement occurs (Figures 5C,E) when the neurorobot was rewarded, but they show no significant changes when the neurorobot is not rewarded (Figures 5D,F). The PMC1 and PMC2 average firing rates increased from 4.21 to 9.63 Hz and from 4.34 to 10.59 Hz, respectively (Figures 5C,E). However, the average rate changed from 3.89 to 3.95 Hz in Figure 5D and from 3.91 to 4.02 Hz in Figure 5F.

## 7. Discussion and future work

Robotic applications seem to be the future of our society due to a rapid evolution in advanced technologies. Many developers, researchers, and scientists have focused on physical robots (Breazeal and Aryananda, 2002; Hegel et al., 2006) that mimic human emotions by recognizing the emotional content in a human's speech. On the other hand, we have paid more attention to how the brain and its related biological processes, and cognition, are involved in human-robot interactions. The development of our VNR has emphasized the integration of ESP as a reward into a virtual neurorobotic system.

During our human emotional speech classification performance, seven English speaking humans were able to classify German speakers' emotions (fear, anger, happy, and sad) with an accuracy of 88.6%, which provided a benchmark for our emotional speech classification system. Since there was a 98.6% accuracy between the “happy” and “sad” utterances, these were chosen to be used as a spoken reward in our virtual neurorobotic application.

Using the Berlin emotional speech database, the average pitch (extracted from our system) between two emotional classes (“happy” and “sad”) and groups of speakers (male and female) was significantly different. This confirmed that RAPT was a successful method for extracting the pitch out of every sample. Using the libSVM model, our offline mode system performance had an accuracy of 95.3% and our live recognition system performance attained similar accuracy by classifying the different emotions correctly 98.7% of the time.

Based on the system performances, we created a scenario where natural speech was used as a reward during a simple exercise. Our emotional speech processing system accurately distinguished between two classes of emotions, happy and sad, and provided a more natural and efficient way for training a child-like robot. ESP was translated to the presented VNR example to encourage or discourage the neurorobot's actions. The plasticity of the synaptic connections was shown as an increase in the synaptic strengths (between VC1 and PMC1, and VC2 and PMC2) and in the firing rates of PMC1 and PMC2 when a reward was given. On the other hand, the absence of reward showed no significant synaptic strengths nor firing rates increase in the concerned regions. These results give a preliminary evaluation when a spoken reward was used as an external stimulus into a neuromorphic brain architecture. In terms of applications, an emphasis was placed on robotic and automated agents. However, our system is by no means limited to that specific application.

Overall, we described how our spoken reward system was successfully used as reinforcement learning and allow our neurorobot to learn a simple exercise and make ideal choices based on visual cues. The ability to monitor and modify simulations in real-time was incredibly useful, especially when we further improve to spiking networks to a larger scale. More importantly, this could demonstrate another step towards multi-scale visualization of neural simulations in a virtual environment.

We are also currently working on the emotional classification system to accurately determine between additional classes in a live environment. Furthermore, the creation of additional virtual robotic scenarios could allow varying degrees of rewards, such as more emotions, and additional external cues, such as facial recognition. Ultimately, we plan create a biologically-realistic emotional classification system that extracts pitch features using a spiking cochlear model, and classifies emotions using a more biologically realistic neural network.

### Conflict of interest statement

The authors declare that the research was conducted in the absence of any commercial or financial relationships that could be construed as a potential conflict of interest.
